# MLS-Net: An Automatic Sleep Stage Classifier Utilizing Multimodal Physiological Signals in Mice

**DOI:** 10.3390/bios14080406

**Published:** 2024-08-22

**Authors:** Chengyong Jiang, Wenbin Xie, Jiadong Zheng, Biao Yan, Junwen Luo, Jiayi Zhang

**Affiliations:** State Key Laboratory of Medical Neurobiology, MOE Frontiers Center for Brain Science, Institutes of Brain Science, Institute for Medical and Engineering Innovation, Department of Ophthalmology and Vision Science, Eye & ENT Hospital, Fudan University, Shanghai 200032, China; 22111520017@m.fudan.edu.cn (C.J.); xiewenbin80@163.com (W.X.); 20301050022@fudan.edu.cn (J.Z.); biaoyan@fudan.edu.cn (B.Y.)

**Keywords:** MLS-Net, ASSC, eye movements, multimodal signal

## Abstract

Over the past decades, feature-based statistical machine learning and deep neural networks have been extensively utilized for automatic sleep stage classification (ASSC). Feature-based approaches offer clear insights into sleep characteristics and require low computational power but often fail to capture the spatial–temporal context of the data. In contrast, deep neural networks can process raw sleep signals directly and deliver superior performance. However, their overfitting, inconsistent accuracy, and computational cost were the primary drawbacks that limited their end-user acceptance. To address these challenges, we developed a novel neural network model, MLS-Net, which integrates the strengths of neural networks and feature extraction for automated sleep staging in mice. MLS-Net leverages temporal and spectral features from multimodal signals, such as EEG, EMG, and eye movements (EMs), as inputs and incorporates a bidirectional Long Short-Term Memory (bi-LSTM) to effectively capture the spatial–temporal nonlinear characteristics inherent in sleep signals. Our studies demonstrate that MLS-Net achieves an overall classification accuracy of 90.4% and REM state precision of 91.1%, sensitivity of 84.7%, and an F1-Score of 87.5% in mice, outperforming other neural network and feature-based algorithms in our multimodal dataset.

## 1. Introduction

Sleep and wakefulness are self-regulatory states of the body that are closely associated with numerous physiological processes. These processes include cognition, learning and memory, brain homeostasis, and various diseases [1,2]. Sleep disorders, such as sleep apnea [3], insomnia [4,5], cataplexy, and narcolepsy [6,7], represent one of the serious problems of modern society. Animal experiments have provided invaluable insights into the mechanisms underlying sleep regulation and sleep disorders [5,8,9]. Given the substantial reliance on rodent models in sleep research, developing rodent ASSC capable of discerning wake–sleep states for rodents would be greatly helpful for the diagnosis of disease and practice in preclinical sleep research [8]. However, rodent ASSC faces two major challenges: first, there is an urgent need to explore an appropriate model that can achieve high performance; second, the impact of multi-modal signal integration on the performance of automatic scoring algorithms is unclear.

The approaches applied to rodent ASSC can be delineated into two principal modalities [10]. The one is a conventional feature-based statistical machine learning approach, while the other involves a deep neural network. Feature-based statistical machine learning feeds the pre-defined feature vectors into vector-based classifiers such as support vector machine (SVM) [11,12], random forest (RF) [13], linear regression (LR), eXtreme Gradient Boosting (XGBoost) [14], linear discriminant analysis (LDA) [15], and so on. Deep neural networks process raw sleep biosignals directly, feeding them into well-designed neural networks for feature extraction and stage classification. This approach has gained popularity due to its outstanding performance [16]. Despite the widespread application of deep neural networks, they present significant limitations. First, their generalization capabilities are often inadequate, particularly when applied to datasets involving animals from different experimental studies [17]. Second, the inherent complexity of these networks can result in the unintended capture of noise, which exacerbates overfitting [18,19]. This issue not only reduces the accuracy of the models but also impedes their practical application in real-time sleep scoring and sleep intervention [20]. This results in significant variability in accuracy, with reported performance ranging from 71.0% to 92.2% [21]. In contrast, feature-based approaches enable users to have clear information about the sleep characteristics underpinning the scoring process with a low energy cost [21]. However, they typically analyze each segment of data independently based on the pre-defined features and prior knowledge, without considering the spatial–temporal context. This limitation prevents them from extracting deeply concealed nonlinear characteristics from the signals, leading to less accurate classification [22].

Currently, rodent ASSC primarily relies on EEG and/or EMG signals due to the small size of rodents [23], which limits the acquisition and application of multi-modal physiological signals. Although alternative studies in humans have demonstrated the benefits of incorporating multi-modal signals [21] such as electromyogram (EOG), electrocardiogram (ECG) [24], respiration, snoring [25], and oxygen saturation [26] for improved classification performance [20,27], this approach has not been widely adopted in rodent research. Eye movements (EMs), as a fundamental indicator in distinguishing REM from NREM sleep [28], have significant potential to enhance the performance of ASSC in rodents, yet their application remains rare. Moreover, despite the extensive use of rodents like mice in preclinical sleep studies, there is a notable lack of publicly available multi-modal physiological datasets for rodents. Unlike human ASSC, which benefits from numerous open datasets for model validation, existing mouse sleep datasets, such as AccuSleep [29], UTSN-L [30], and SPINDLE [17], are limited to EEG/EMG data. The absence of a common multi-modal dataset contributes to discrepancies in published results across different research teams [23], highlighting the critical need for establishing an open multi-modal physiological dataset for mice to accelerate integrative physiology as well as behavioral studies.

**Figure 1:**
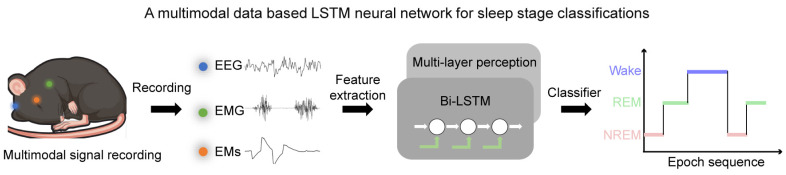
The framework of a MLS-Net model using multimodal physiological signals. In MLS-Net model, the multimodal signals, including EEG, EMG, and EM signals, were collected from sleeping mice. The feature vectors of time–domain and frequency–domain were fed into bi-LSTM and then bi-LSTM using the feed-forward multi-layer perceptron (MLP) to compute the output probability of each sleep stage to identify the target of the signal.

To solve these dilemmas, we designed a neural network, MLS-Net, that combines the strengths of neural networks and feature extraction based on multi-model physiological signals for automated sleep staging in mice (Figure 1). The core innovation of MLS-Net lies in: (1) a tailor-designed bi-LSTM’s proven capability in handling sequences and its effectiveness in capturing the spatial–temporal nonlinear characteristics inherent in consecutive sleep states during sleep; and (2) employs time–domain and frequency–domain expert features of multimodal signals, especially EMs, as inputs, which provides deterministic expert knowledge. This design can effectively address the issue of traditional deep neural networks experiencing a marked decline in classification accuracy for specific sleep stages, such as the REM stage [17]. Our studies, conducted on a robust dataset, show that MLS-Net achieves an impressive overall accuracy of 90.4% among other neural networks and feature-based algorithms. Notably, it excels in REM sleep detection, achieving a precision of 91.1%, a sensitivity of 84.7%, and an F1-Score of 87.5% in mice. These results underscore the advantages of using multimodal data and advanced neural architectures in sleep research. The main contributions of this work are summarized as follows:

(1) We propose a novel rodent automatic sleep classifier, MLS-Net, that demonstrates superior performance compared with other classifiers, achieving an overall accuracy rate of 90.4%.
(2) Our proposed model is based on a multimodal paradigm that takes multimodal signals (EEG, EMG, and EMs) as input. The employment of EM signals enhances the detection of REM sleep, achieving a precision of 91.1%, sensitivity of 84.7%, and an F1-Score of 87.5% in mice.
(3) We will open-source this multimodal dataset to foster broader research in sleep studies.
(4) Our system achieves an inference time of 86.1 s for 24 h sleep recordings, which show strong potential for real-time applications.

## 2. Related Works

### 2.1. Different Approaches for ASSC in Human Dataset

Feature-based statistical machine learning approaches leverage pre-defined features as input. Classification accuracies vary significantly among the different classifiers reported in the literature, ranging from 70% to 94% [31]. Sanders et al. [32] proposed that a linear discriminant analysis (LDA) classifier achieved an average accuracy of 75% in ASSC using average power and cross-frequency coupling features extracted from EEG. Ra Boostani et al. [33] carried out a comparative review of several machine learning classification techniques used in ASSC and found that the RF classifier achieved a higher accuracy of 87.49% in healthy humans compared with KNN, SVM, and linear discriminant analysis (LDA). Similarly, Rahman et al. [34] reported that SVM (89.9%) and RF (90.2%) performed consistently better in terms of overall accuracy compared with RUSBoost on the SleepEDF dataset. Yoon et al. reported the encouraging performance of KNN with an accuracy of 98.65% using multi-modal signals, which has been adopted for sleep monitoring in a home-adapted device [35].

In recent years, deep neural networks, such as convolutional neural networks (CNNs) [36,37], recurrent neural networks (RNNs) [38], long short-term memory (LSTM) [39], deep belief networks (DBNs), and combinations of different network architectures [40,41], have been widely used in ASSC due to their capacity to handle large datasets effectively. For example (Table 1), Pei et al. [16] achieved an accuracy of 83.15% in ASSC using CNN and the gated recurrent unit (GRU) network on multichannel EEG data from the human sleep database. Integrating GNN with bidirectional GRU, Einizade et al. [42] reported an accuracy of 83.8% using multi-modal signals from the Sleep-EDF dataset. Awais et al. [43] designed a fusion model by integrating a DCNN with a SVM, achieving an accuracy of 93.8% in neonatal sleep stage classification using facial expression video data. The most impressive accuracy was reported by Mousavi et al. [44], who proposed an attention-based CNN-LSTM approach for sleep–wake detection using acceleration and heart rate variability and achieved 94.8% accuracy. However, the difficulty of collecting acceleration and heart rate data makes it unsuitable for further application.

### 2.2. Different Approaches for ASSC in Mice

The use of animal models is critical in preclinical research for circadian rhythm studies, disease modeling, and sleep monitor equipment development [23,53]. While ASSC has made significant progress in clinically utilizing multi-modal physiological signals, research in mice predominantly relies on single-modal signals such as EEG or EMG alone or a combination of EEG and EMG [23]. Various machine learning algorithms have been applied to rodent sleep classification, yielding promising results as shown in Table 2. While the success of these approaches has varied, the most effective methods have achieved approximately 92% accuracy in categorizing the three states of sleep–wake behavior [54]. For instance, Gross et al. [55] developed an open auto-scorer system based on complex Boolean logical decisions, achieving an accuracy of 80.24% using EEG and EMG signals in rats. Brankačk et al. [15] employed a linear discriminant analysis (LDA) classifier to predict vigilance states from 73 variables extracted from EEG data, attaining an accuracy of 89% in 10 mice. Rempe et al. [56] applied a hybrid approach combining principal component analysis (PCA) and a Naive Bayes classifier (NBC) for sleep classification using EEG and EMG signals. Although they achieved an overall accuracy of 90%, the accuracy for detecting REM sleep was only 70%. Svetnik et al. [13] compared the performance of the SVM algorithm with that of a deep neural network in mice, finding that the SVM achieved a comparable accuracy of 81%, only slightly lower than the 83% accuracy obtained with the deep neural network. In another study, Fraigne et al. [54] developed a novel ensemble learning approach, SleepEns, built on the Time Series Ensemble, which achieved 90% accuracy relative to expert scorers in mice. In contrast to the use of a single classifier, Gao et al. [12] proposed that employing multiple classifier systems is a more effective approach to improving the accuracy of automated sleep scoring.

Recent advancements have leveraged neural networks that utilize large amounts of training data and computational power to generate accurate predictions, eliminating the need for preliminary feature extraction. Exarchos et al. [57] applied a CNN-based method, achieving a mean accuracy of approximately 85–90% for REM sleep detection. Tezuka et al. [30] developed UTSN-L, which processes single-channel EEG signals using a CNN combined with a LSTM network to incorporate historical information, reaching an overall accuracy of 90% in the mouse dataset. Yamabe et al. [58] generated the MC-SleepNet model using the same stratagem as UTSN-L and obtained 96.6% accuracy on a large-scale dataset. Pawan K. Jha et al. [59] created SlumberNet, which leverages the ResNet architecture for sleep scoring using EEG and EMG signals and excels with 97% overall accuracy. Although those state-of-the-art models achieved impressive accuracy in intra-lab validation, it also raises the concern of generalizing those models. Three publications [13,17,60] have described scoring algorithms validated across different datasets or species. To address the generalization challenges of ASSC in animals, Miladinović et al. [17] developed SPINDLE, a hybrid deep learning model incorporating a hidden Markov model (HMM) and CNN architecture. Empirical evidence indicates that SPINDLE effectively classifies sleep stages with an accuracy range of 93–99% while exhibiting robustness across diverse laboratory datasets. Similarly, Svetnik et al. [13] employed a CNN-based approach for sleep–wake scoring in non-human primates and other animals, achieving test accuracies of 75% for macaques, 83% for mice, 78% for rats, and 73% for dogs. Furthermore, Kam et al. [60] proposed the WSN, which utilizes wavelet-transformed images of mouse EEG/EMG signals. Their method achieved an accuracy of 86%, validated through a leave-one-subject-out (LOSO) approach on their dataset, as well as validation with an external dataset.

**Table 2:** Summary of different sleep-stage algorithms in mice.

Type	Classifier	Dataset	Channels	Validation	Accuracy	Ref.
Machine learning algorithm	Auto-Scorer	6 rats	EEG, EMG	Intra-lab	80.24%	2009 [55]
	LDA	10 mice	EEG	Intra-lab	89%	2010 [15]
	PCA/NBC	12 mice	EEG, EMG	Intra-lab	90%	2015 [56]
	RF	Rhesus macaques,	EEG, EMG and ACT	Across-species	Macaques: 66%	2020 [13]
		Mice,			Mice: 81%	
		Rat,			Rat: 77%	
		Dog			Dogs: 64%	
	SleepEns	28 mice	EEG, EMG	Intra-lab	90%	2023 [54]
Neural network	HMM+CNN	14 mice and 8 rats	EEG, EMG	Cross-Lab	93–99%	2019 [17]
		(SPINDLE dataset)				
	CNN+LSTM	Large-scale dataset (4200 recordings);	EEG, EMG	Intra-lab	96.60%	2019 [58]
		Small dataset (14 recordings)				
	CNN	Rhesus macaques,	EEG, EMG and ACT	Across-species	Macaques: 75%	2020 [13]
		Mice,			Mice: 83%	
		Rat,			Rat: 78%	
		Dog			Dogs: 73%	
	CNN	7 mice	EEG, EMG	Intra-lab	85–90%	2020 [57]
	CNN-LSTM	216 recordings (UNST-L dataset)	EEG	Intra-lab	90%	2021 [30]
	CNN	20 mice from intra-lab;	Image dataset from EEG, EMG	Intra and cross-lab	Intra-lab: 86%	2021 [60]
		AccuSleep dataset			Cross-lab: >85%	
	Residual	5 mice	EEG, EMG	Intra-lab	97%	2024 [59]
	Networks					
	Pre-defined features and LSTM-MLP	7 mice from intra-lab;	EEG, EMG, EMs	Intra-lab	Intra-lab: 90.4%	Our work
		AccuSleep dataset		cross-lab	Cross-lab: 89.9%	

**Notation:** LDA: linear discriminant analysis; PCA: principal component analysis; NBC: naive bayes classifier; RF: random forest; residual networks: based on a two-dimensional convolutional layer (Conv2D); ACT: locomotor activity; HMM: hidden Markov model.

### 2.3. Comparisons of MLS-Net with Prior Studies

In general, the poor generalization and suitability of feature-based statistical machine learning approaches result in inconsistent performance in ASSC [31], even when the same classifier is applied to the same dataset. Moreover, these approaches analyze each epoch independently, thereby failing to capture the time series information and the inherent rules of sleep transitions. Deep neural networks can partially address these shortcomings due to their enormous power to extract hidden information [21]. However, the non-interpretability of the model prediction and the longer computational times hinder the application of these models in real-time hardware implementations [20].

Our proposed MLS-Net integrates the strengths of feature-based approaches and deep neural networks. It uses the deterministic features extracted from multimodal physiological signals as input, which are then fed into a bi-LSTM network. These deterministic features enhance the interpretability of the model’s performance. The LSTM network addresses the limitations of traditional machine learning methods (analyze each epoch independently) by processing time-series data and considering sleep transition rules. MLS-Net achieves optimal performance on our dataset with significantly reduced training time compared with other models. Most importantly, the lightweight nature of MLS-Net makes it highly feasible for real-time application in the future.

## 3. Materials and Methods

### 3.1. Animal

Male young C57Bl/6 J mice were purchased from Shanghai Jiesjie Laboratory Animal Co., LTD, China, and housed in rooms at 25 ± 1 °C and 50–70% humidity, under a 12/12 hr light/dark cycle (light on at 7 a.m.), with free access to food and water. For polysomnographic recording, the mice were surgically implanted with EEG and EMG according to previously established procedures [61]. All experiments were conducted in accordance with the National Institutes of Health Guide for the Care and Use of Laboratory Animals and were approved by the Animal Care and Use Committee of Shanghai Medical College of Fudan University.

### 3.2. Multimodal Dataset Design and Implementation

For multimodal physiological signal recording, 7 healthy mice were recorded, and each mouse was recorded for 3 days according to the following procedure. A polysomnographic recording device was designed as previously described [61]. For EEG recording, two stainless steel screws were inserted on the top of left and right skulls at anteroposterior (AP) +1.5 mm, mediolateral (ML) +1.5 mm, and AP −3.5 mm, ML 3 mm, respectively. For EMG recording, two EMG electrodes were inserted into the neck musculature of the mice. Since freely moving mice close their eyes while sleeping, we cannot use cameras [62] or electrode eye coils to record Ems [63]. In our previous work, we innovatively proposed a novel eye movement tracking technique [61]. In brief, a miniature, strong magnetic rod was implanted in the conjunctiva of mice, and a magnetic displacement sensor was integrated into the polysomnographic device. The EMs of mice drive the sensor to cut through the magnetic field lines generated by the magnetic rod. The resulting change in the magnetic signal indicates the EMs of mice during sleep. Using this technique, we successfully achieved the simultaneous collection of EEG, EMG, and EM signals during mouse sleep.

To annotate the sleep–wake states of each epoch, raw EEG and EMG traces were visually inspected offline and scored into three vigilance states: wakefulness, NREM sleep, and REM sleep using SleepSign software (Version 2.0, Kissei Comtec, Nagano, Japan), followed by manual calibration by trained experts. The sleep states were segmented into non-overlapping consecutive 4 s epochs. Specifically, wakefulness epoch was characterized by increased EMG activity and low EEG amplitude. NREM sleep epoch was identified by delta-dominant EEG (0.5–4 Hz) and low EMG power. REM sleep epoch was defined based on theta-dominant EEG (6–9 Hz) and low EMG power.

### 3.3. MLS-Net Model

**Figure 2:**
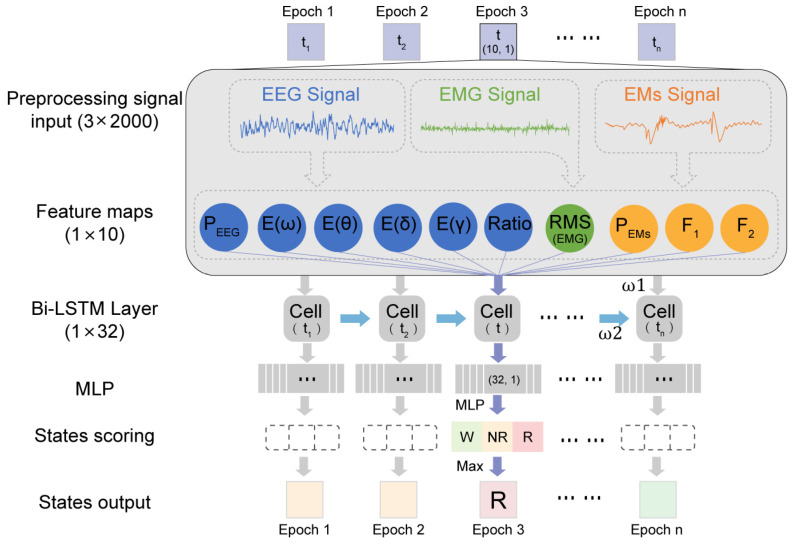
Illustration of MLS-Net model.

Our proposed MLS-Net algorithm consists of input preprocessing, feature extraction, bi-LSTM, multi-layer perception, and scoring output, as shown in Figure 2. Before implementing ASSC, a notch filter was applied to eliminate 50 Hz noise in EEG, EMG, and EMs. The EMG signal was subsequently processed using a Butterworth bandpass filter with a frequency range of 30–70 Hz to effectively eliminate significant electrical noise. Subsequently, extreme outliers in EEG and EMs caused by motion artifacts and EMG contamination were removed as follows. We defined the extreme lower tail percentile at 0.01 th and extreme higher tail percentile at 99.99 th in EEG and EM signals. The data exceeding the thresholds were set to the threshold values and then normalized using the following formula. Subsequently, the time and frequency domain features were extracted from outlier removal and normalized multimodal physiological signals. The feature vectors were fed into a bi-LSTM layer, which was a sequence-processing neural network that incorporated past states (i.e., epochs) into the classification of present states and captured temporal dependencies. The bi-LSTM layer comprises 16 units in each direction, totaling 32 units. The outputs from the bi-LSTM layer are then fed into a multi-layer perceptron (MLP), followed by a softmax layer, which generates class probabilities.

$${Signal}_{Normalized}=\frac{Signal-\mu_{Signal}}{\sigma_{Signal}}$$

*Signal* refers to multi-modal signals. *μ_Signal_* is the mean of the raw signal, and the *δ_Signal_* is the standard deviation of the raw signal. This formula is general for EEG, EMG, and EMs by changing the subscript “*Signal*’ to the respective signal type.

The neural network models were trained using an Intel Core i7-11800H processor, 16 GB of memory (8 GB × 2), and an NVIDIA GeForce RTX 3060 GPU.

### 3.4. Expert-Knowledge-Based Feature Extraction

For pre-defined features, all methods tested in this paper start with the identical feature extraction step, where a wide range of features were computed from the pre-processed EEG, EOG, and EMG signals (linear time and frequency domains and non-linear time–domain features). Frequency domain features of EEG signals were performed using Fast Fourier Transform (FFT) algorithms. The energy of EMG, which carries information about muscle activity, was also calculated. The frequency of EMs was determined according to our previous research [61]. A total of 10 features were derived for feature-based analysis, including the power of regular waves (0.5–4 Hz, 6–9 Hz, 8–12 Hz, and 52–70 Hz), the root mean-squared of EMG, the mean amplitude of EEG and EMs, and theta/delta EEG power ratio.

(1) Amplitude and Power Calculation for EEG Signals:
  (1) $${Power}_{signal}(\omega )=\sum_{t=0}^{T}\left|{F\left(\omega ,t\right)|}^{2}for\omega \epsilon [0.5Hz,100Hz\right]$$
  where *F(w, t)* is the FFT of the signal at time t and frequency ω, and *T* is the total duration. This formula is general for EEG, EMG, and EMs by changing the subscript “signal” to the respective signal type.

(2) Spectral Power Features for EEG:
  (2) $$P_{EEG}\left(band\right)=\frac{\sum_{t=start}^{end}\left[\sum_{f=f_{low}}^{f_{high}}|{F\left(f,t\right)|}^{2}\right]}{end-start+1}\;$$
  where band specifies the frequency band (e.g., *P_EEG_(δ)* ϵ [0.45 Hz, 4 Hz], E(θ) ϵ [6 Hz–9 Hz], upper E(θ) [7 Hz, 8.5 Hz], E(α) ϵ [8 Hz, 12 Hz], P_EEG_(ω) ϵ [0 Hz, 30 Hz], upper E(γ) ϵ [52 Hz, 70 Hz]). The upper E(θ) and upper E(γ) have been proven to be effective for discriminating between sleep and wake [15]. *f_low_* and *f_high_* are the boundaries of the frequency band, and start and end define the time window.

(3) Theta/Delta Ratio and Sleep State EM Features:
  (3) $${Ratio}_{\theta /\delta }=\frac{P_{EEG}\;(theta)}{P_{EEG}\;(delta)}\;,\;\;F_{EMs\;}\left(state\right)=f_{EMs}(A_{state})\mathrm{for}\mathrm{state}\epsilon (\mathrm{wake, REM})$$
  where *P_EEG_ (theta)*and *P_EEG_ (delta)* are power computations in the theta (6–9 Hz) and delta (0.45–4 Hz) bands, respectively, and *A_state_* denotes the threshold values (mean ± 1.96 × std.) of EMs during different periods (wakefulness or REM). *F_EMs_(state)* represent the frequency of EMs exceeding *A_wake_* and *A_REM_* thresholds in the current epoch.

(4) The root mean squared (RMS) of EMG:
  $$RMS=\sqrt[{2}]{\frac{1}{n}\;\sum_{i=1}^{N}{x_{i}}^{2}}$$

*N* represents the total number of data points in the EEG signal. *x_i_* denotes the *i*-th data point of the signal.

### 3.5. Evaluation

**Figure 3:**
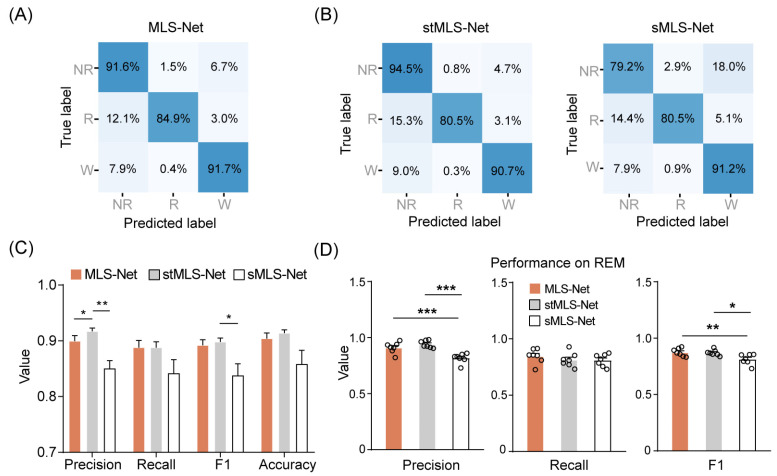
Performance of the MLS-Net for sleep-wake classification. (**A**) Confusion matrix of MLS-Net evaluated on the test set. (**B**) Confusion matrix of stMLS-Net and sMLS-Net evaluated on the test set. Spatio–temporal MLS-Net model (stMLS-Net) and spatial MLS-Net model (sMLS-Net) were variants based on MLS-Net model. Each element in the matrices indicates the percentage of correct matching between experts (True label) and each model (Predicted label). The abbreviations used are as follows: wake (W), eye movement sleep (R), non-eye movement sleep (NR). **(C)** Overall performance of MLS-Net, stMLS-Net and sMLS-Net models. (**D**) Precision, sensitivity (recall), and F1-Score of MLS-Net, stMLS-Net and sMLS-Net models for REM stage classification. Empty circle in the histogram indicates individual result in cross-validation. One-way ANOVA followed Dunnett’s post hoc analysis. Data were presented as mean ± SEM. * *p* < 0.05, ** *p* < 0.01, *** *p* < 0.001.

In order to evaluate the training and judgment accuracy of different models, all algorithms should be developed using the same channels, trained on the same pre-clinical dataset, and validated with the same procedure. In our algorithm, the effectiveness of different algorithms was validated by leave-one-out cross-validation (LOOCV). Out of all the “k” recordings derived from 7 mice, a single record was retained as the validation data for testing the model, and the remaining k-1 records were used as training sample. The LOOCV process was repeated k times and yielded k results, with each of the k records used exactly once. The average of k results was deemed to be a single estimation for each algorithm. Using these strategies, all records were used for both training and validation, so that the evaluation bias due to single training could be effectively avoided without compromising the stability and reproducibility of the algorithms. Five different evaluation measures are used to evaluate the performance of the proposed method, including the confusion matrix, accuracy, precision, sensitivity (recall), and F1-Score, as shown in Figure 3 and Table 4 [64].

(4) $$Accuracy=\frac{TP+TN}{TP+FN+FP+TN}\%Precision=\frac{TP}{TP+FP}\%$$

(5) $$\;Recall=\frac{TP}{TP+FN}\%F1-Score=2\frac{Precision\times Recall}{Precision+Recall}$$

In this context, TN, FN, FP, and TP refer to true negatives, false negatives, false positives, and true positives, respectively. TN represents the number of sleep stages that were incorrectly classified as corresponding to the labeled sleep stages. FN denotes the number of sleep stages that were inaccurately identified as other stages when they should have been classified as the correct stage. FP refers to the number of sleep stages that were erroneously classified as labeled stages when they were not. TP indicates the number of sleep stages that were correctly classified in accordance with their labeled stage.

The coefficient of variation (CV) within groups is calculated using the following formula:

$$CV=\frac{\sigma }{\mu }\times 100\%$$

In this formula, *σ* is the standard deviation of each accuracy for all-fold cross-validation, *μ* is the mean of the performance accuracy.

### 3.6. The Construction of Rebalance Dataset Using Data Augmentation

To address the class imbalance issue, we performed data augmentation by repeating the minority class samples as suggested in previous studies [65]. The raw epoch sequences comprise varying durations for wake, NREM, and REM epochs. Initially, we calculated the mean length of epochs within each continuous class. Subsequently, we trimmed all epochs exceeding this mean length to the calculated value. We then increased the number of samples for the minority classes, expanding them to match the mean duration of the wake, NREM, and REM classes in the input sequence. This approach enabled us to construct an augmented dataset, referred to as the balanced dataset, ensuring that all sleep stages are equally represented in the training set. The augmentation process is illustrated in Figure 6A.

## 4. Result

### 4.1. Dataset

From the animal experiments, a small-scale sleep recording dataset was compiled. A total of seven recordings from seven healthy wild-type mice were conducted. Each recording lasted three days, with data from the last 24 h incorporated into the dataset. As shown in Table 3, the multimodal dataset comprises 151,200 epochs, including 82,449 wake epochs (54.5%), 56,286 NREM epochs (37.2%), and 12,465 REM epochs (8.2%). The proportions of sleep architecture are consistent with previous reports [66]. Each epoch includes one channel of EEG, EMG, and EM signals, simultaneously collected with a duration of 4 s at a 500 Hz sample rate. Prior to feature extraction, preprocessing was conducted, which included 50 Hz noise filtering and the removal of outliers in artifact-contaminated epochs, following the predefined rules outlined in the methods section. For inner-cross validation, the dataset was split into training and validation sets, with 85.7% of the sleep samples used for training and the remaining 14.3% for validation.

### 4.2. The Performance of MLS-Net and Its Variants

We designed the MLS-Net to process multi-modal signals from our constructed dataset. To minimize the variance of predictions across different mouse recordings, we employed a cross-validation approach using seven separate MLS-Net trained on a Leave-One-Out Cross-Validation (LOOCV) method. This approach ensured that six animal recordings were used for training, while the remaining one, which was never seen by the model during training, was used for validation. Figure 3A shows the average confusion matrix comparing the true and predicted sleep labels for the input test set after the 120 th iteration of training on the MLS-Net model. Our proposed MLS-Net model achieves favorable diagonal coefficients in its confusion matrix, with an accuracy of 91.6% for NREM, 84.9% for REM, and 91.7% for wake, respectively. and yielded an overall decoding precision of 89.9%, a mean sensitivity (recall) of 88.8%, an F1-Score of 89.2%, and an overall accuracy of 90.4% (Table 4, Figure 3C).

The issue of network structure is crucial for understanding the trade-offs between model complexity and its practical application, especially in real-time systems such as sleep state analysis. To explore the appropriate structure of the model, we further generated two variants on the basis of MLS-Net. In the spatial MLS-Net model (sMLS-Net), the pre-defined features were removed and replaced by a two-layer CNN for spatial feature extraction from EEG, EMG, and EM signals, followed by an MLP layer and a softmax layer. The spatio–temporal MLS-Net model (stMLS-Net) reserved the pre-defined features, which were concatenated with spatial features extracted from CNN as input. The series information was also captured by a bi-LSTM neural network. These are then processed through a MLP, and finally, a softmax layer outputs three targets. These two classifiers were trained and tested using the identical data partitions (six training sets versus one testing set) of our dataset.

Compared with MLS-Net, the stMLS-Net model shows a competitive performance on wake (90.7%) and NREM (94.5%), but the sMLS-Net model had lower accuracy both in NREM (79.2%) and REM (80.5%), as shown in Figure 3B. The overall performance of MLS-Net and stMLS-Net is comparable in terms of precision, sensitivity (recall), and F1-Score, with both models outperforming the sMLS-Net model in these metrics (Figure 3C). This result demonstrated the importance of the LSTM layer in MLS-Net, as the sMLS-Net, which lacks the LSTM layer, shows poor performance, especially in REM sleep classification (Figure 3D).

### 4.3. Performance Comparison of Various Classifiers

**Figure 4:**
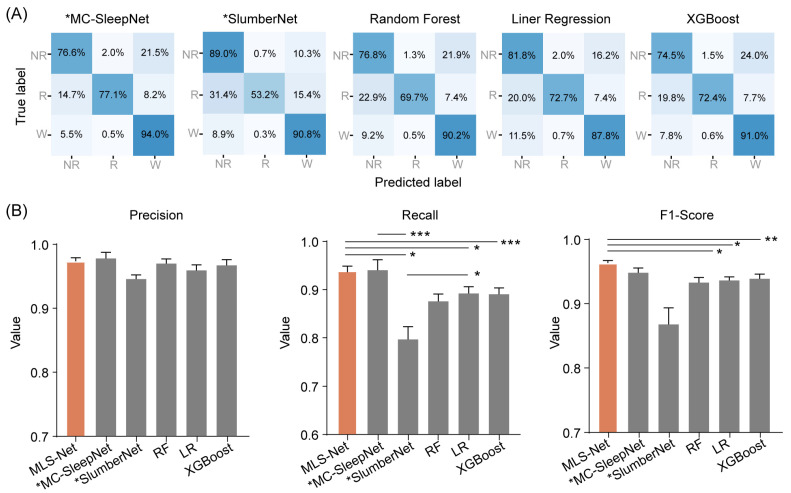
The performance of different classifiers on REM classification. (**A**) Shows the confusion matrix of MC-SleepNet, SlumberNet, random forest (RF), linear regression (LR), and eXtreme Gradient Boosting (XGBoost). The state-of-the-art models MC-SleepNet and SlumberNet were denoted with an asterisk (*MC-SleepNet, *SlumberNet). Each element in the matrices indicates the percentage of correct matching between experts (True label) and each model (Predicted label). The abbreviations used are as follows: wake (W), eye movement sleep (R), non-eye movement sleep (NR). (**B**) shows the performance comparison of precision, sensitivity (recall), and F1-Score for REM classification. The orange bar indicates the performance of MLS-Net model and the gray bars indicate the performance of other models. One-way ANOVA followed Dunnett’s post hoc analysis. Data were presented as mean ± SEM. * *p* < 0.05, ** *p* < 0.01, *** *p* < 0.001.

To evaluate the performance of MLS-Net, we conducted a comparative analysis against five other classifiers. Among these, two represent state-of-the-art models based on neural networks, specifically MC-SleepNet [58] and SlumberNet [59], while the remaining three are machine learning models: random forest (RF), linear regression (LR), and XGBoost. To ensure a fair comparison, all models were trained and tested on the same dataset, following identical preprocessing steps. Notably, MC-SleepNet and SlumberNet were well-deigned to process EEG and EMG signals, whereas the other three models were meticulously adjusted to accommodate EM signals as input. The predictive performance of these models is summarized in Figure 4A and Table 4.

**Figure 5:**
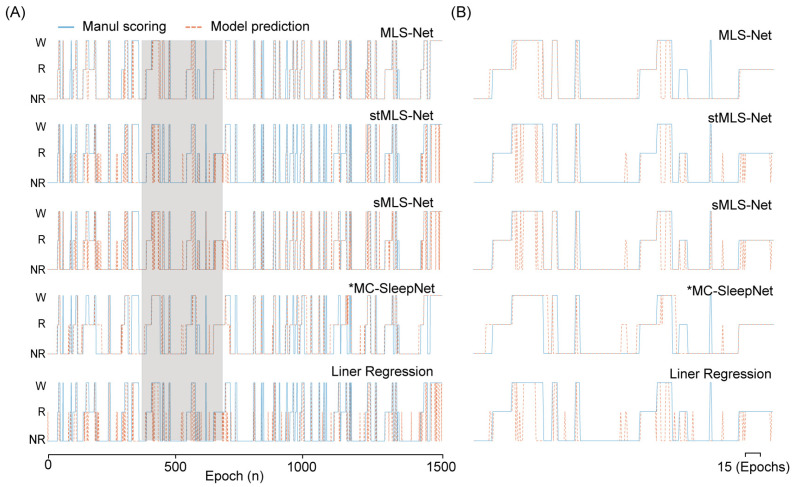
The output hypnogram predicted by different models compared with manual scoring. (**A**) shows example hypnograms of one subject. The solid blue line and yellow dashed line denote the hypnograms depicted by the proposed model and an expert, respectively. (**B**) was the enlargement of hypnogram in the shielding area (**A**).

The confusion matrix reveals that both algorithms exhibit excellent performance in classifying the wake but perform poorly in distinguishing NREM and REM stages, especially in the case of SlumberNet and feature-based machine learning algorithms (Figure 3D). From the confusion matrix, we can see that the primary source of misclassification for NREM stages is their erroneous attribution to the wake stage, while misclassification of REM stages mainly arises from their incorrect attribution to NREM stages, as shown in the hypnograms in Figure 5A,B. In terms of overall accuracy, the MLS-Net model achieved the highest accuracy (89.4%), followed closely by the MC-SleepNet (89.0%). The SlumberNet, RF, LR, and XGBoost models achieved accuracies of 86.5%, 83.3%, 73.1%, and 83.6%, respectively (Table 4). In addition to performance, we also compared the training time consumed by each classifier. We found that the MLS-Net model had a significantly faster training time of 86.1 s per 120 iterations over the same training set than its two variants (stMLS-Net, sMLS-Net) and MC-SleepNet (608.9 s). In contrast, SlumberNet requires a longer training duration, up to 3718.4 s for each LOOCV.

Further inspection shows that MLS-Net achieved a precision of 92.1%, a sensitivity (recall) of 83.4%, and an F1-Score of 87.3% for REM sleep (Figure 4B). The sensitivity of MLS-Net in REM classification outperforms SlumberNet and traditional machine learning algorithms (Figure 4B). This improvement can be attributed to the utilization of EM signals, which enhance the discrimination between REM and NREM sleep stages.

### 4.4. The Performance of Different Classifiers on Balanced Dataset

**Figure 6:**
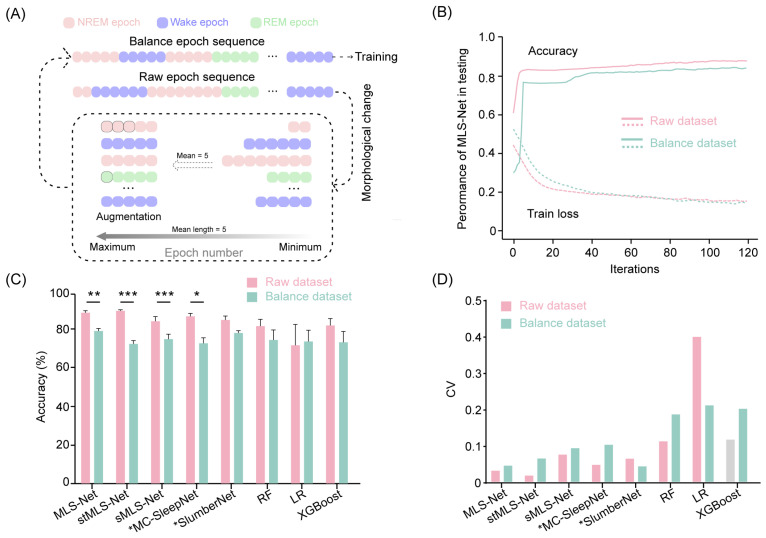
Accuracy comparison cross-models in raw dataset and balanced dataset. (**A**) The generic workflow of data augmentation. (**B**) Accuracy and loss curves of the training and testing on raw dataset and balanced dataset. (**C**) The scoring accuracy of different models for all folds in cross-validation. Two-sided paired Student’s t-test between raw dataset and balanced dataset for each model. Data were presented as mean ± SEM. * *p* < 0.05, ** *p* < 0.01, *** *p* < 0.001. (**D**) The coefficient of variation (CV) of accuracy for all folds in cross-validation.

The sleep stages identified by the expert occur with widely differing frequencies [65] (e.g., wake: 54%, REM: 8.2%, NREM: 37.2% in our dataset). Such differences in the frequencies of classes (class imbalance) can potentially hinder the performance of classifiers and limit further advancements in sleep staging algorithms [65,67]. To address this issue, we employed a data augmentation technique by duplicating minority class samples in the sleep epoch sequence (Figure 6A). In the balanced dataset, all samples had an equal opportunity to compensate for the minority classes. Next, we trained and tested the performance on both rebalance datasets. After 120 iterations on the training dataset, the overall accuracy of the MLS-Net, sMLS-Net, and SlumberNet models on the balanced dataset decreased by 9.2% (from 90.4% to 81.2%), 9.48% (from 85.8% to 76.4%), and 6.9% (from 86.5% to 79.6%), respectively (Figure 4B). Although stMLS-Net and MC-SleepNet exhibited comparable performance to the MLS-Net model on the standard dataset (Tabel 4), their accuracy on the balanced dataset dropped even more significantly, with declines of 15.7% (from 91.4% to 75.7%) and 14.4% (from 89.0% to 74.6%), respectively (Figure 4B). These results suggest that both the spatio–temporal MLS-Net (stMLS-Net) and MC-SleepNet networks are more susceptible to imbalanced tasks in this work. Moreover, MLS-Net consistently outperformed the other algorithms with relatively lower variance for each model of the LOOCV cross-validation (Figure 6D).

### 4.5. The Influence of EM Signals on MLS-Net Model

**Figure 7:**
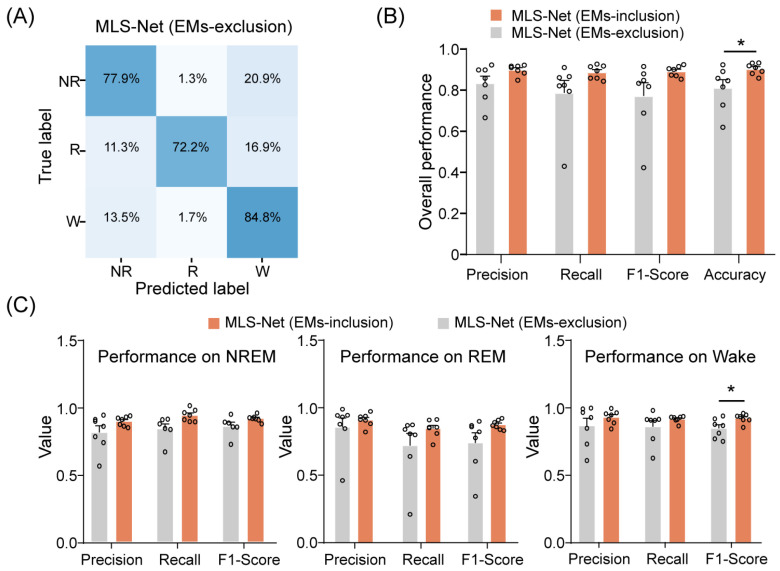
The performance of MLS-Net models after EMs-exclusion. (**A**) The confusion matrix of MLS-Net performance on the EMs-exclusion dataset. (**B**) Overall accuracy of MLS-Net performance across all folds of cross-validation. (**C**) Precision, sensitivity (recall), and F1-Score for individual stage classification following EMs-exclusion. Empty circle in the histogram indicates individual result in cross-validation. Two-sided Student’s *t*-test. Data were presented as mean ± SEM. * *p* < 0.05.

Overall, our proposed MLS-Net model demonstrates performance that is equal to or superior to that of state-of-the-art models such as MC-SleepNet, SlumberNet, and other machine learning algorithms on both standard and imbalanced datasets. One possible reason for this enhanced performance is the use of EM signals. To evaluate the contribution of EM signals to the performance of MLS-Net, we performed EM-exclusion testing, where features extracted from EMs were replaced with a null mask. The class-specific accuracy was evaluated using a mean confusion matrix and overall accuracy metrics, as illustrated in Figure 7A,B.

The exclusion of EM signals resulted in agreement rates of 77.9% and 72.2% for REM and NREM sleep, respectively, between predicted classifications and true labels. An increased incidence of erroneously categorizing REM and NREM epochs as wake epochs was observed, as depicted in Figure 7A. Consequently, the overall accuracy of sleep state scoring significantly decreased from 90.4% to 81.2% when EM signals were excluded. (Figure 7B). A closer investigation has shown that precision, sensitivity (recall), and F1-Score all decreased for individual stages after EMs-exclusion (Figure 7C). Specifically, the F1-Score for wake significantly declined from 92.7% to 84.6% after EMs-exclusion, as illustrated in Figure 7C. These results indicate that incorporating EM signals can significantly enhance the performance of the MLS-Net by providing additional information during training.

### 4.6. External Validation of MLS-Net Model

To evaluate the generalizability of MLS-Net, we conducted an external validation using the AccuSleep mouse EEG/EMG sleep dataset (https://doi.org/10.17605/OSF.IO/PY5EB, accessed on 22 July 2024). This dataset includes sleep recordings from five healthy mice (mouse01–mouse05), each consisting of two complete 24 h polysomnography (PSG) records. The PSG data comprise one EEG and one EMG signal, sampled at 512 Hz, with sleep states annotated by experts every 2.5 s. We utilized a continuous 24 h segment from each mouse to validate the performance of the MLS-Net model. To ensure a fair comparison, all data were processed within the Pytorch framework, and the parameters of the baseline model were maintained as previously specified.

**Figure 8:**
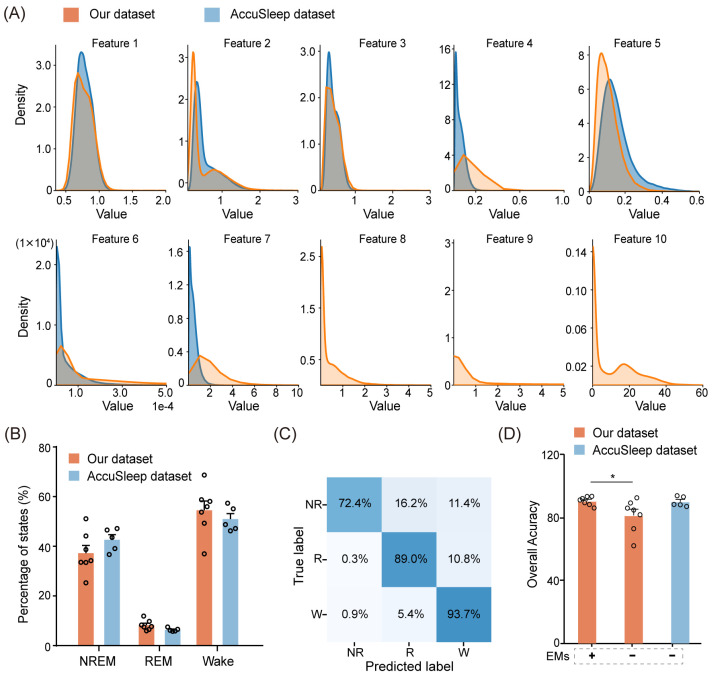
Performance of MLS-Net on an external dataset. (**A**) The distribution of pre-defined features extracted from each epoch in our dataset (yellow) and the external dataset (blue). (**B**) A comparison of sleep architecture between our dataset and the external dataset. (**C**) The confusion matrix demonstrating the performance of MLS-Net on the external dataset. (**D**) Overall accuracy of MLS-Net performance on the external dataset. The symbols ‘+’ and ‘−’ indicate testing on the EMs included and EMs excluded datasets, respectively. Empty circle in the histogram indicates individual result in cross-validation. One-way ANOVA followed Dunnett’s post hoc analysis. Data were presented as mean ± SEM. * *p* < 0.05.

Since the AccuSleep dataset does not include EM signals, we left the corresponding EM parameters (Feature 8, 9, and 10) blank, as shown in Figure 8A. Apart from the features extracted from EM signals, the distributions of the multimodal signal features extracted from the external dataset were similar to those extracted from our dataset, as shown in Figure 8A. From a sleep perspective, we further compared the proportions of NREM, REM, and wake epochs between the external dataset and our dataset. The external dataset had proportions of 42.6% for NREM, 6.4% for REM, and 50.9% for wake, which were analogous to the proportions in our dataset (average 37.2% for NREM, 8.2% for REM, and 54.5% for wake) in Figure 8B.

The performance of MLS-Net in the AccuSleep dataset was shown in the confusion matrix and overall accuracy against consensus manual scores. As shown in Figure 8C, the agreement between MLS-Net output and human score on the AccuSleep dataset has reached 93.7% and 89.0% for wake and REM stage recognition, respectively. The mean accuracy on the AccuSleep dataset was 89.9%, which is slightly lower than the accuracy of 90.4% achieved on our dataset (Figure 8D). The accuracy of 89.9% in the AccuSleep dataset, exceeding 80.6% achieved in the EMs-exclusion test on our dataset, can be attributed to two main factors. Firstly, the high quality of EEG and EMG signals in the external dataset enabled the model to capture sufficient information for parameter optimization. Secondly, the performance of a neural network is correlated with the amount of training data [28]. Therefore, the shorter duration of sleep state epochs (2.5 s/epoch vs. 4 s/epoch) in the external dataset allowed for a greater number of epochs to be extracted from the same amount of sleep data for training. In summary, MLS-Net successfully withstood external data validation, thereby further enhancing the model’s generalizability.

## 5. Discussion

### 5.1. Hybrid of Feature-Based and Neural Network Methodology

Over the past 30 years, automatic sleep scoring has seen significant advancements. Compared with feature-based methodologies, deep neural networks demonstrate remarkable efficacy in extracting latent information from raw physiological signals. However, its “black box” nature makes the information difficult to characterize and translate into the clear, interpretable features used in feature-based approaches [21]. The lack of interpretability in results and prolonged computational times are the main drawbacks that could limit acceptance by end users [68]. In contrast, feature-based approaches provide clear insights into the sleep characteristics considered during scoring. Traditional feature-based approaches also present challenges, as they analyze each input segment independently and do not fully exploit the temporal sequence information inherent in sleep–wake transitions. The bi-LSTM neural network, with its forget gate structure, widens the usage of past information and is better suited for time series data [64]. In this work, we leverage the strengths of both feature-based approaches and bi-LSTM neural networks to develop the MLS-Net model, which utilizes deterministic expert knowledge to enhance the performance of neural networks. The performance of MLS-Net is validated by LOOCV in mice. This internal cross-validation ensures that data from the test set does not appear in the training set, thereby enhancing the model’s generalizability to unfamiliar datasets.

The overall decoding precision, sensitivity, F1-Score, and accuracy of MLS-Net at the testing set are 89.9%, 88.8%, 89.2%, and 90.4%, respectively. After training with a cross-validated approach, we performed a head-to-head comparison and found that the MLS-Net model demonstrates superior performance over the state-of-the-art MC-SleepNet [58], SlumberNet [59], and traditional machining learning models with higher accuracy and the lowest CV when using the same channels (Figure 6). Due to the relatively small proportion (5–10%) of REM epochs within the entire sleep–wake cycle, accurately identifying REM sleep poses a significant challenge. Our findings demonstrate that MLS-Net achieved an 84.9% agreement with expert scorers on REM epoch classification, while MC-SleepNet, SlumberNet, random forest (RF), linear regression (LR), and XGBoost achieved agreement rates of 77.1%, 53.2%, 69.7%, 72.7%, and 72.4%, respectively. Meanwhile, the promising and stable performance of MLS-Net was validated using an external dataset, where it maintained an accuracy of over 89% even without the use of EM signals. The notable performance in REM sleep classification can be attributed to the incorporation of deterministic features extracted based on expert knowledge from multi-modal signals and the temporal information captured by the bi-LSTM layers.

### 5.2. EM Signals during REM Sleep Contributes to Sleep Classification

EMs in sleep were originally characterized as falling into the non-rapid eye movement sleep (NREM) and rapid eye movement sleep (REM) categories [69]. REM sleep is characterized by frequent EMs, whereas NREM sleep is marked by the relative absence of such movements. This phenomenon is highly conserved across different species, from humans to rodents [61,70]. Previous studies reported that the average frequency of EMs in mice was about 0.90, 0.25, and 0.05 Hz during wake, REM, and NREM sleep, respectively [71]. Thus, EMs could be a potential indicator to distinguish between different stages in mice. One non-invasive method for detecting EMs is through EOG recording, which has been shown to effectively enhance the performance of sleep classification algorithms in clinical settings [34]. However, the intricate technical challenges have impeded its implementation in small animals [72]. Our previous work [61] enabled the simultaneous collection of EEG, EMG, and EM signals from freely moving mice. In this study, we deigned MLS-Net based on three modalities of signal fusions (EEG/EMG/EMs). By performing EMs-exclusion testing, we found that EMs significantly improved the performance of MSL-Net in discriminating sleep status with high accuracy, which was paralleled with counterpart models.

### 5.3. Limitations and Future Perspectives

Although the MLS-Net model had substantial agreement with sleep experts, there are important limitations to consider. While we tested the model with an external mouse sleep dataset, the robustness and generalizability of MLS-Net have not yet been validated for human use. Human PSG records involve a more complex classification into multiple sleep stages compared with rodents, which may pose greater challenges [23]. Much research has been done on improving human EEG classification as well [12]. MLS-Net capitalizes on the strengths of neural networks in capturing temporal sequence information while incorporating predefined features extracted from multimodal signals such as EEG, EMG, and EMs from mice. Due to the significant differences in sleep architecture and feature distributions between mice and humans, our findings cannot be directly extended to human studies. However, given that multi-modal electrophysiological data can be collected not only in mice but also in humans [73], adapting the MLS-Net model for human datasets could involve several potential modifications. These may include adjusting the frequency ranges used in feature extraction to better align with human EEG frequencies or fine-tuning the memory capacity of LSTM layers to more effectively capture the long-term dependencies characteristic of human sleep patterns. Secondly, the outstanding performance of the MLS-Net model was validated on a small-scale dataset, but its performance on large-scale datasets remains unknown. In theory, a larger amount of training data would lead to a better training effect for the deep learning method [28]. Additionally, our study was limited in exploring potential variations in accuracy due to factors such as age, behavior, gender, or genetic variability among mice.

Given the inherent advantages and limitations of various preprocessing methodologies, an optimal ASSC system may be derived from a judicious amalgamation of different techniques [10]. Future research in deep learning is expected to prioritize the development of simpler models for ASSC that maintain high accuracy while optimizing portability and efficiency. Efforts should also focus on extracting channel-specific information and identifying micro-stages within NREM and REM sleep without compromising algorithmic stability. Moreover, enhancing model compatibility is crucial to facilitate its application in sleep classification for the pathological states of rodents, potentially aiding in the screening and identification of physiological markers for sleep-related mental illnesses in preclinical research.

## 6. Conclusions

This study designed the MLS-Net model that combines the strengths of neural networks and feature extraction for rodent ASSC using multimodal biological signals (e.g., EEG, EMG, EMs). By leveraging explicit feature inputs, the proposed MLS-Net model effectively mitigates the overfitting commonly associated with neural networks and addresses the low accuracy issues typically encountered in feature-based machine learning methods. The MLS-Net model has a runtime of only 86.1 s for 24 h sleep recordings, which is significantly faster than the several hundred to thousands of seconds required by other networks. The time-saving property without compromising its efficiency makes MLS-Net suitable for real-time sleep scoring applications in preclinical research. Moreover, the algorithm offers flexibility for users who may wish to customize it to better suit their specific needs. Potential modifications include using alternate features, adjusting artifact detection criteria during preprocessing, or incorporating additional signals to enhance performance.

## Figures and Tables

**Table 1:** Summary of different sleep-stage algorithms in human.

Type	Classifier	Dataset	Channels	Accuracy	Ref.
Machine learning algorithm	Dendrogram multi-class SVM	15 subjects	EOG, EMG, EEG	88%	2015 [45]
	RF	Sleep–EDF dataset and CAP sleep dataset	EEG	87.49%	2017 [33]
	SVM	Sleep-EDF, Sleep-EDFX and ISRUC-Sleep databases	EOG	SVM: 89.9%	2018 [34]
	RF			RF: 90.2%	
	RUSBoost			RUSBoost:88.6%	
	KNN	10 subjects	PSG	98.65%	2022 [35]
	SVM;	10 subjects	EEG, ECG, PPG, EOG	SVM: 70.05%	2023 [46]
	RF;			RF: 70.75%	
	SleepBoost; CatBoost; GBoost; RF	20 recordings from Sleep-EDF dataset	EEG, EOG, EMG	86.30%	2024 [47]
Neural network	DNN	MIT-BIH database (18 subjects)	EEG, ECG	73.70%	2018 [48]
	RNN + CNN	Montreal Archive of Sleep Studies (200 subjects)	EEG, EOG, EMG.	87.10%	2019 [49]
	MLP	30 s segments from 19	EEG	82.53%	2020 [50]
	CNN	42 recordings from University Hospital of Leuven	EEG	64%	2020 [51]
	CNN + GRUs	SHHS (700 subjects)	EOG, EMG, EEG	83.15%	2020 [16]
	DCNN + SVM	19 Chinese neonates at the Children’s Hospital of Fudan University	Vedio, facial expressions	93.80%	2021 [43]
	CNN + LSTM	Clinical dataset	HRV + acceleration	94.50%	2021 [44]
	GNN+bi-GRU	MASS;	EOG, EMG, ECG, EEG	86.70%	2023 [42]
		SleepEDF dataset		83.80%	
	Traditional machine learning and deep learning techniques.	100 recordings from ISRUC and 197 recordings from Sleep-EDFx dataset	EEG, EMG, ECG	ISRUC dataset:	2024 [52]
				80.30%	
				Sleep-EDF dataset:	
				81%	

**Notation:** RF: random forest; RUSBoost: random under-sampling boosting; SVM: support vector machine; CatBoost: categorical boost; RNN: recurrent neural network; CNN: convolutional neural network; MLP: multi-layer perceptron; LSTM: long short-term memory; DNN: deep neural network; DCNN: deep convolutional neural network; GRUs: gated recurrent units. ECG: electrocardiogram, EEG: electroencephalogram, EMG: electromyogram, EOG: electrooculogram, HRV: heart rate variability. PSG: polysomnogram.

**Table 3:** The distribution of sleep stages in the dataset.

Stage	Total Epochs	Percentage (%)	Training Set (%)	Test Set (%)
Wake	82,449	54.5	85.7 (6/7)	14.3 (1/7)
REM	12,465	8.2		
NREM	56,286	37.2		

**Table 4:** Performance comparison of the different models.

Algorithms	Dataset	EM-Exclusion	Balance	Precision (%)	Recall (%)	F1-Score (%)	Accuracy (%)	Time (s)
**MLS-Net**	Our dataset	No	No	89.9	88.8	89.2	90.4	86.1
		No	Yes	74.2	79.5	81.5	81.2	
		Yes	No	80.8	76.8	78.4	80.6	
	AccuSleep Dataset	--	No	90.0	85.0	87.1	89.9	
**stMLS-Net**	Our dataset	No	No	91.6	88.9	90.0	91.4	3073.3
			Yes	78.4	72.7	74.9	75.7	
**sMLS-Net**	Our dataset	No	No	84.3	83.6	83.9	85.8	479.8
			Yes	74.1	75.4	74.7	76.4	
*MC-SleepNet	Our dataset	No	No	88.5	84.8	86.4	89.0	608.9
			Yes	78.6	70.7	73.5	74.6	
*SlumberNet	Our dataset	No	No	88.0	86.5	86.0	86.5	3178.4
			Yes	80.5	79.8	79.3	79.6	
**RF**	Our dataset	No	No	84.5	78.9	78.3	83.3	2.9
			Yes	78.1	73.4	75.3	68.2	
**LR**	Our dataset	No	No	83.9	80.8	82.2	73.1	10.7
			Yes	76.3	72.0	73.8	67.7	
**XGBoost**	Our dataset	No	No	73.4	59.5	48.0	83.6	22.1
			Yes	76.5	73.4	74.4	67.3	

**Notation:** AccuSleep dataset was an external dataset (https://doi.org/10.17605/OSF.IO/PY5EB accessed on 22 July 2024). EMs-exclusion means whether the EMs signals were removed during the training sets. AccuSleep dataset was devoid of EM signals, so this section was left unfilled. The balance means whether the data augmentation were conducted. All performance metrics (precision, sensitivity (recall), F1-Score, and accuracy) are averaged values across LOOCV validation. All models were tested on both balanced and imbalanced datasets. The accuracy values for the imbalanced dataset (raw dataset) are highlighted in bold. Time of each model indicates the compute time after 120 iterations, with each classifier trained on six recordings.

## Data Availability

The dataset and code utilized in this study are openly accessible. Specifically, the MLS-Net model code referenced in this article is available on Kaggle at the following link: https://www.kaggle.com/models/jiangchengyong/mls-net (accessed on 30 May 2024). Additionally, the multimodal physiological dataset constructed from mice is also uploaded on Kaggle and can be accessed here: https://www.kaggle.com/datasets/chenruilin245/sleepdata (accessed on 30 May 2024). Furthermore, the publicly available AccuSleep dataset, which was analyzed in this study, can be found at: https://doi.org/10.17605/OSF.IO/PY5EB (accessed on 30 May 2024).
